# COVID-19: An Update Regarding the Quest for Finding an Effective Cure

**DOI:** 10.7759/cureus.9010

**Published:** 2020-07-05

**Authors:** Fahed S Bangash, Gulalei Saeed, Palwasha Shahab, Aneeqa Waheed

**Affiliations:** 1 Internal Medicine, Hull University Teaching Hospitals NHS Trust, Hull, GBR; 2 Internal Medicine, Shifa International Hospital, Islamabad, PAK; 3 Medicine, Riphah International Hospital, Islamabad, PAK; 4 Neurology, Lady Reading Hospital, Peshawar, PAK

**Keywords:** corona virus, corona pandemic, vaccine, covid-19, sars-cov-2

## Abstract

The coronavirus disease (COVID-19) is a global pandemic. COVID-19 is caused by a novel coronavirus known as severe acute respiratory syndrome coronavirus-2 (SARS-CoV-2), which was identified at the end of 2019 in a cluster of pneumonia cases in Wuhan, China. It has likely affected everyone in the world either directly or indirectly, financially or through social isolation measures. There are now many publications about its etiology, epidemiology, investigations, and clinical presentation. Therefore, the medical community has a much better understanding of the disease as compared to a few months ago. There is no effective, safe treatment for COVID-19. There are many guidelines, clinical trials, and information on various media platforms that hinder the tracking of developments in this rapidly evolving situation. In this review, we provide a detailed update on various emerging treatment options and analyze the results of recent trials. This review also discusses current progress on finding a COVID-19 vaccine.

## Introduction and background

Coronaviruses are a large family of medium-sized, enveloped positive-stranded ribonucleic acid (RNA) viruses that can cause illness in humans and animals. The name 'coronavirus' derives from the crown-like appearance on the electron micrographs [1]. Its impact on humans can vary from asymptomatic infection or minor illness like the common cold to severe pneumonia leading to severe acute respiratory syndrome (SARS). Thouzgh rare, animal coronaviruses can spread to humans, as evidenced by the SARS coronavirus and Middle East respiratory syndrome (MERS) coronavirus. A study showed that in December 2019, five patients in China who were managed for pneumonia had an unknown coronavirus strain, which had 79.0% and 51.8% nucleotide identity with the sequence of SARS-CoV and MERS-CoV, respectively [2]. The World Health Organization (WHO) published its first disease outbreak news on January 5, 2020, about pneumonia cases of unknown cause surfacing in Wuhan city [3]. Later, on January 12, the genetic sequence of the virus was publicly shared. The virus was declared a public health emergency of international concern on January 30; then, it was declared a pandemic on March 11 [4]. As of this writing, five million cases have been confirmed, and 347,000 deaths have been reported to the WHO [5].

**Figure 1:**
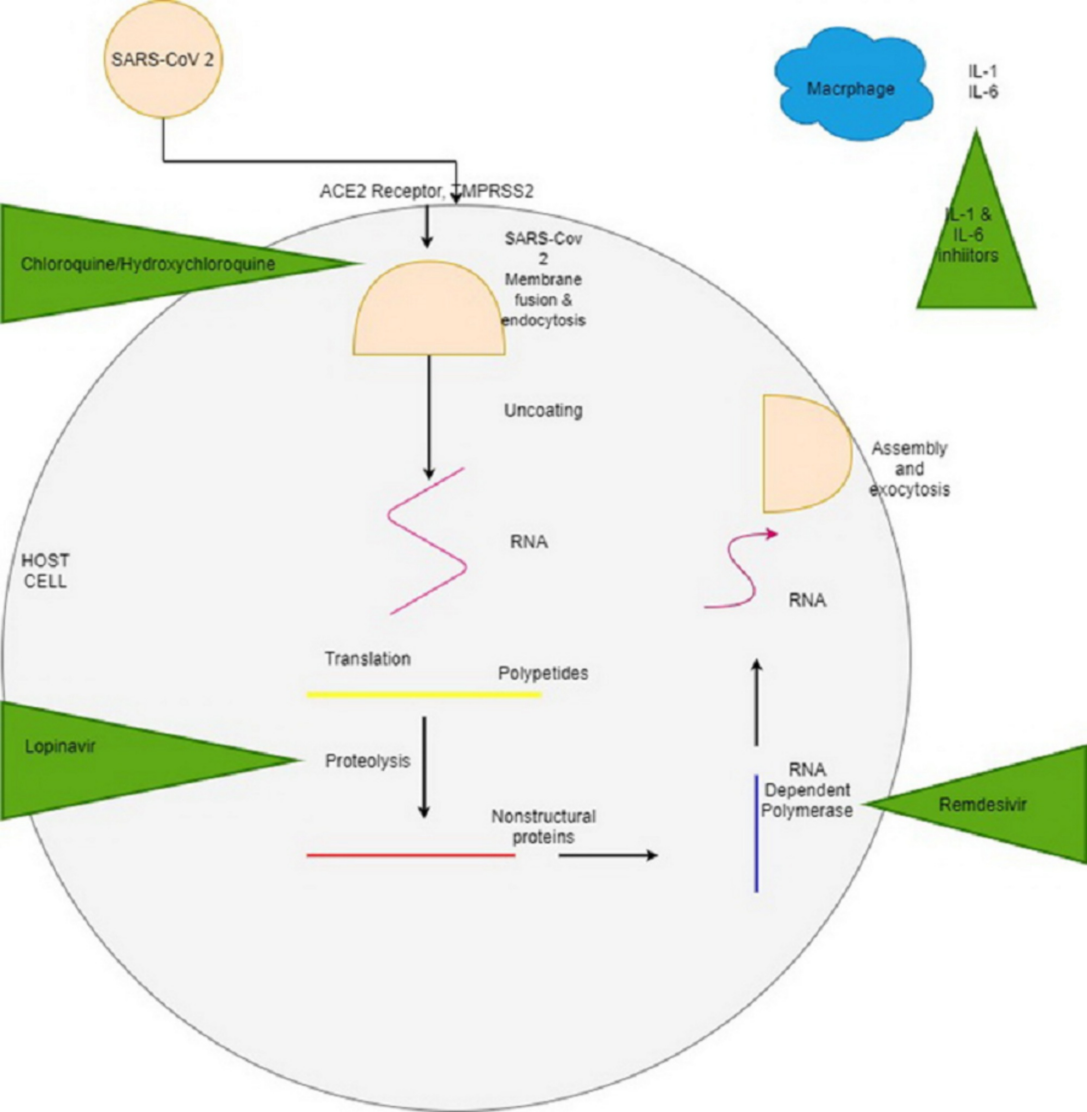
SARS-CoV-2 infecting host cell SARS-CoV-2: severe acute respiratory syndrome coronavirus-2; ACE2: angiotensin-converting enzyme 2; TMPRSS2: transmembrane protease serine 2; IL: interleukin; RNA: ribonucleic acid

Novel coronavirus disease (COVID-19) is caused by the severe acute respiratory syndrome-coronavirus-2 (SARS-CoV-2), which binds angiotensin-converting enzyme 2 (ACE2) receptors and gains entry into the cell via transmembrane protease serine 2 [6]. Once viral RNA is inside the host cell, it uses cellular material for translation and proteolysis to form key proteins like RNA-dependent RNA polymerase, RNA helicase, and other structural proteins [7]. These components then make viral structure components, eventually leading to exocytosis of further viral copies, which then, in turn, invade other cells (Figure 1).

There are currently hundreds of clinical trials in progress to find a cure for COVID-19. In these trials, many therapeutic options are under review, mostly based on the previous experience with SARS and MERS [8]. Therapeutic options include antimalarial medications, antiviral medication (such as those used for HIV), and some modes of passive immunity (Table 1). In these unprecedented circumstances, it is difficult to find effective preventive and curative therapy in a short time as the process of clinical trials is lengthy. This review article provides an update on the various emerging treatment options, trials underway, and current progress of vaccines for COVID-19.

**Table 1:** Emerging treatment options for COVID-19 along with their mechanism of action COVID-19: coronavirus disease; IFN: interferon; HIV: human immunodeficiency virus; RNA: ribonucleic acid; IL, interleukin; JAK: Janus kinase; BCG: Bacille Calmette-Guerin

Drug/Treatment name	Mechanism of action
Chloroquine/ hydroxychloroquine	Antimalarial: interferes with hemoglobin utilization by parasites, raises internal pH of parasitic vesicles.
	Antiviral: pH change at cell membrane surface, inhibits viral fusion
Azithromycin	Antibacterial: inhibits RNA-dependent protein synthesis
	Proposed antiviral effects: induction of IFN-stimulated genes, attenuating viral replication
Remdesivir	Inhibits viral RNA-dependent RNA polymerase
Lopinavir	Antiretroviral protease inhibitor (HIV type 1 aspartate protease inhibitor)
Ritonavir	Increases Lopinavir's half-life through the inhibition of cytochrome P450 component
Convalescent Plasma	Antibodies against coronavirus and may suppress viremia
Intravenous immunoglobulin	Provides passive immunity, neutralizes the pathogens, blocks the receptors associated with the target cell, and prevents the pathogen from further damaging the target cell
IL-1 Inhibitors	Antagonist of the IL-1 receptor
IL-6 Inhibitors	Antagonist of the IL-6 receptor
IFN (Alpha, Beta)	Increases phagocytic activity of macrophages
JAK Inhibitors	Selectively inhibits JAK
Stem cell therapy	Mesenchymal stem cells may reduce the pathological changes that occur in the lungs and inhibit the cell-mediated immunologic response
BCG vaccine	Some evidence of induction of innate immune memory

## Review

The management of COVID-19 is evolving quickly as new information is learned. This is a new virus causing a new disease, so, currently, there is no Food and Drug Administration (FDA) approved treatment. Based on previous experience with viruses like SARS and MERS, we have some treatment options which are now in the process of clinical trials. Research regarding the safety and efficacy of potential treatments for COVID-19 is ongoing. The following is a review of the various therapeutic options undergoing clinical trials and an update about ongoing vaccine trials.

Chloroquine, hydroxychloroquine, and combination of azithromycin with chloroquine or hydroxychloroquine

Chloroquine and hydroxychloroquine (HCQ) are oral medications used to treat malaria and autoimmune conditions like systemic lupus erythematosus and rheumatoid arthritis (RA). They also have antiviral effects that are not fully understood. They may impair the release of the virus from the endosome or lysosome because these drugs increase endosomal and lysosomal pH; the release of the virus requires a low pH [9]. By altering cellular pH, the virus is unable to release its genetic material into the cell and replicate [10,11]. Azithromycin is widely used for its antibacterial effects, but it may have antiviral effects from the induction of interferon-stimulated genes, thereby attenuating viral replication. These treatments are associated with QTc prolongation, and combined use may potentiate this adverse effect.

HCQ has the advantage of fewer side effects but the same therapeutic effects as chloroquine. One of the first randomized controlled trials (RCTs) of 150 people in China found no evidence of increased clearance SARS-CoV-2 when HCQ was used in comparison to the standard of care; however, HCQ did improve C-reactive protein quicker than the control group [12].

A multinational registry analysis of the usage of chloroquine or HCQ with or without azithromycin was published on May 22, 2020 [13]. The study used the data from 671 hospitals across the world from December 2019 to April 2020, involving 14,888 patients in the treatment groups using either chloroquine or HCQ and 81,144 patients in the control group. The study found that chloroquine and HCQ both increased mortality in comparison to the control group and increased the frequency of ventricular arrhythmias. Given this study was observational, further RCTs would provide better evidence regarding its safety and efficacy. Recently the Food and Drug Administration (FDA) has cautioned against chloroquine and HCQ use in COVID-19 patients except in a clinical trial.

Remdesivir

Remdesivir is an antiviral nucleotide prodrug of an adenosine analog and is administered intravenously (IV). Remdesivir binds to the viral RNA-dependent RNA polymerase, inhibiting viral replication through premature termination of RNA transcription. It has demonstrated in vitro activity against SARS-CoV-2 [14] and in vitro and in vivo activity (based on animal studies) against SARS-CoV and MERS-CoV [15].

The FDA has given emergency approval for remdesivir use in patients with severe COVID-19 [16]. A preliminary report analyzed data from a small cohort of patients who received at least one dose of remdesivir on a compassionate basis, which showed improvement in 68% percent of patients (36 of 54 patients) [17]. A randomized, double-blinded trial from China showed that patients receiving remdesivir had clinical improvement in 21 days compared to 23 days to improvement for control patients; the difference was not statistically significant. However, patients who received remdesivir within the first 10 days of symptom onset had a median clinical improvement time of 18 days compared to 23 days in the control group [18]. Preliminary data from a recently published double-blinded RCT with 1063 patients showed a median recovery time was of 11 days with an estimated mortality of 7.1% for the remdesivir group while the median recovery time was 15 days with an estimated mortality of 11.9% for the control group [19].

These studies suggest that remdesivir has the potential to reduce mortality but would not suffice as a standalone treatment for COVID-19.

Lopinavir and ritonavir

Lopinavir and ritonavir are in a coformulation working as an antiretroviral protease inhibitor. The replication of SARS-CoV-2 depends on the cleavage of polyproteins into an RNA-dependent RNA polymerase and a helicase. Two proteases are responsible for this cleavage: 3-chymotrypsin-like protease (3CLpro) and papain-like protease. Lopinavir/ritonavir is an inhibitor of SARS-CoV 3CLpro in vitro, and this protease appears highly conserved in SARS-CoV-2 [20]. The ritonavir component inhibits the CYP3A metabolism of lopinavir, allowing increased plasma levels of lopinavir. In a randomized, open-label trial of 199 hospitalized COVID-19 patients, 99 patients received lopinavir-ritonavir, while 100 patients received standard care [21]. Results showed no difference in time to clinical improvement or mortality in comparison to standard care. In a further modified intention to treat analysis, duration to clinical improvement was significant by one median day. The trial was not blinded, and overall mortality was higher among all patients, which could mean that severely ill patients were enrolled in the trial. Further trials are required to determine the efficacy of lopinavir-ritonavir.

Convalescent plasma/IV immunoglobulin

Plasma donated from individuals who have recovered from COVID-19, which contains antibodies to SARS-CoV-2, may help suppress the virus and may modify the inflammatory response. SARS-CoV-2 IV immunoglobulin (IVIG) is a concentrated antibody preparation derived from the plasma of people who have recovered from COVID-19.

Convalescent plasma transfusion (CPT) has been previously used for different infectious diseases. It was used in 2004 for SARS infection, and a study in Hong Kong showed its effectiveness by decreasing mortality, especially when given early in the disease course [22]. CPT has been effective in treating patients with severe pandemic influenza A with significantly lower mortality [23]. One of the earliest systemic reviews of CPT showed that mortality was decreased, viral clearance was enhanced, and clinical symptoms improved [24].

In a retrospective study done at Wuhan Third Hospital, 58 patients with severe COVID-19 received intravenous immunoglobin (IVIG). The study found that when IVIG is used within 48 hours, mechanical ventilator use, duration of hospital stay, and mortality were all reduced. However, prospective RCTs are warranted to confirm these findings [25].

Interleukin-1 inhibitors

The cytokine profile of many patients with moderate to severe COVID-19 overlaps with those seen in macrophage activation syndrome (MAS) and secondary hemophagocytic lymphohistiocytosis (sHLH) [26]. Viruses are known triggers of MAS/sHLH, and high ferritin levels are associated with both MAS and mortality in patients with COVID-19. Endogenous interleukin-1 (IL-1, a pro-inflammatory cytokine) potently induces IL-6 in monocytes and macrophages and is elevated in patients with COVID-19, MAS, and other conditions such as severe chimeric antigen receptor T-cell (CAR-T) mediated cytokine release syndrome (CRS).

Anakinra is a recombinant human IL-1 receptor antagonist. It is approved to treat a variety of inflammatory conditions that range from RA to familial Mediterranean fever. Anakinra is also used off-label for severe CAR-T mediated CRS and MAS/sHLH [27]. However, there are no published studies to date on the use of anakinra in COVID-19 infection or for other novel coronavirus infections.

IL-6 inhibitors

COVID-19 may activate a dysregulated host immune response leading to elevated IL-6 levels. A systematic review and meta-analysis of eight studies showed elevated IL-6 levels (2.9x) in complicated COVID-19 cases in comparison to noncomplicated COVID-19 cases [28]. IL-6 receptor antagonist monoclonal antibodies (e.g., tocilizumab) are being tested in clinical trials in COVID-19 patients for the treatment of virus-induced CRS [29]. Tocilizumab is a biological disease-modifying anti-rheumatic biological drug mostly used for the treatment of mild to moderate RA [30], and it was recently approved in 2017 for treating severe or life-threatening CAR-T-induced CRS [31].

In a nonrandomized open-label study, 21 patients with severe COVID-19 were treated with tocilizumab. Researchers found a significant decrease in oxygen requirement along with improvement in chest X-ray findings and clinical symptoms [32]. Currently, there are no data from randomized clinical trials, and more evidence is required.

Interferons

Interferons are antiviral cytokines explored as a potential treatment of COVID-19 due to their in vitro and in vivo antiviral properties. However, lack of benefit when interferons were used in other coronavirus infections (e.g., MERS, SARS) and the absence of clinical trial results in COVID-19 make it an unusual choice of treatment given its adverse effects. An open-label randomized trial in Hong Kong showed that a triple therapy of antivirals (ribavirin, lopinavir-ritonavir, and interferon-Beta-1b) in comparison to lopinavir-ritonavir was safe and superior when given early in the course of the disease. It increased viral clearance and reduced inpatient stay time in mild to moderate COVID-19 cases [33]. As this study was based on a combination of antivirals, separate studies evaluating interferons are required to further evaluate the safety and efficacy of interferons on its own.

Janus kinase inhibitors

Janus kinase (JAK) inhibitors work by inhibiting the JAK-signal transducer and the activator of the transcription pathway. Baricitinib is a JAK inhibitor with potent anti-inflammatory activity against interferon-associated inflammation. It is FDA-approved to treat RA [34], and it may have an antiviral effect by inhibiting the receptor-mediated endocytosis of the virus into host cells [35]. A pilot study was done to assess the safety and efficacy of baricitinib in COVID-19, which showed that it was safe and improved clinical parameters [36]. The pilot study was limited in that it was a small, open-label nonrandomized study of 12 patients, but it led the way for further studies.

Stem-cell therapy

Mesenchymal stem cells (MSCs) have regenerative properties and can differentiate into diverse cell lineages. MSCs have generated considerable interest among researchers whose work has offered intriguing perspectives on cell-based therapies for various diseases. MSCs are being investigated in a clinical trial for COVID-19, which focuses on the safety and efficiency of MSC therapy for pneumonia patients infected with SARS-CoV-2 [37]. MSCs may reduce the pathological changes that occur in the lungs and inhibit the cell-mediated inflammatory response.

Bacille Calmette-Guerin vaccine

The Bacille Calmette-Guerin (BCG) vaccine is used in countries where tuberculosis (TB) is common, and in countries where TB is less prevalent, BCG is typically only used for children at high risk. COVID-19 has affected more than 200 countries, but its impact is not as high (in mortality) in countries where the BCG vaccination is used according to the WHO COVID-19 Situation Report 125 [38]. This has led researchers to test the BCG vaccine. A pilot RCT showed that a BCG vaccination before influenza vaccination had a better antibody response against the H1N1 influenza strain [39]. Another study showed that COVID-19-associated mortality in countries that use BCG is 5.8 times lower than countries not using routine BCG vaccination [40]. Another study suggests that BCG vaccination has little to no correlation with COVID-19 [41].

Quest for a vaccine

There is currently no vaccine approved for the prevention of COVID-19. Vaccines are currently in development; however, as with any clinical vaccination trial, the formulation must go through a standard process that may last 12 to 18 months. According to the WHO draft landscape of COVID-19 vaccines (as of May 22, 2020), 10 vaccines are currently at the clinical evaluation stage undergoing Phase I/II trials in humans (Table 2) [42]. Moreover, there are 112 vaccines in preclinical evaluation [42]. These vaccines include messenger RNA (mRNA) and DNA platform vaccines, adenovirus vector vaccines, and inactivated virus vaccines. This section will briefly outline the 10 current vaccine candidates currently approved by the WHO for clinical evaluation.

**Table 2:** Ten vaccines approved by the World Health Organization. Adapted from WHO draft landscape of COVID-19 candidate vaccines [42]. NIAID: National Institute of Allergy and Infectious Diseases; RNA: ribonucleic acid; LNP: lipid nanoparticle; mRNA: messenger RNA; IND: investigational new drug.

Platform	Type of candidate vaccine	Developer	Current stage of clinical evaluation/regulatory status of Coronavirus candidate
Nonreplicating Viral Vector	Adenovirus Type 5 Vector	CanSino Biological Inc./Beijing Institute of Biotechnology	Phase II ChiCTR2000031781 Phase 1 ChiCTR2000030906
RNA	LNP-encapsulated mRNA	Moderna/NIAID	Phase II (IND submission) Phase 1 NCT04283461
Inactivated	Inactivated	Wuhan Institute of Biological Products/Sinopharm	Phase I/II ChiCTR2000031809
Inactivated	Inactivated	Beijing Institute of Biological Products/Sinopharm	Phase I/II ChiCTR2000032459
Inactivated	Inactivated + alum	Sinovac	Phase I/II NCT04352608
Nonreplicating Viral Vector	ChAdOx1	University of Oxford	Phase I/II NCT04324606
Protein subunit	Full-length recombinant SARS CoV-2 glycoprotein nanoparticle vaccine adjuvanted with Matrix M	Novavax	Phase 1/2 NCT04368988
RNA	3 LNP-mRNAs	BioNTech/Fosun Pharma/Pfizer	Phase I/II 2020-001038-36
DNA	DNA plasmid vaccine with electroporation	Inovio Pharmaceuticals	Phase I NCT04336410
Inactivated	Inactivated	Institute of Medical Biology , Chinese Academy of Medical Sciences	Phase 1

A Randomized, Double-Blinded, Placebo-Controlled Phase II Clinical Trial for Recombinant Novel Coronavirus (2019-nCOV) Vaccine (Adenovirus Vector)

CanSino Biological is collaborating with the Beijing Institute of Biotechnology and has the only vaccine in a Phase II trial. The vaccine uses an adenovirus vector and is set to be tested in 375 healthy adults, with 125 people in the control group. The trial will assess adverse reactions and levels of COVID-19 neutralizing antibodies within 14 days, along with antibodies against the coronavirus spike protein at day 28. Participants will be monitored via follow-up for a maximum of six months [42-43].

Phase I, Open-Label, Dose-Ranging Study of the Safety and Immunogenicity of 2019-nCoV Vaccine (mRNA-1273) in Healthy Adults

This is a Phase I, open-label, dose-ranging clinical trial in men and nonpregnant women aged 18 years or older in good health and who meet all eligibility criteria. This trial will assess the safety, reactogenicity, and immunogenicity of mRNA-1273 manufactured by ModernaTX, Inc (Cambridge, MA). The vaccine uses an RNA platform and is being given in two doses, with the second dose 28 days after the first [42, 44].

Evaluation of the Safety and Immunogenicity of Inactivated Novel Coronavirus Pneumonia (COVID-19) Vaccine (Vero Cells) in Healthy Population Aged Six Years and Above: A Randomized, Double-blind, Placebo Parallel-Controlled Phase I/II Clinical Trial

This inactivated vero cell vaccine is currently undergoing clinical trials in China. It was developed by the Wuhan Institute of Biological Products/Sinopharm, and the study timeline is April 11, 2020, to November 10, 2021 [42].

Evaluation of the Safety and Immunogenicity of Inactivated Novel Coronavirus (2019-CoV) Vaccine (Vero Cells) in Healthy Population Aged Three Years and Above: A Randomized, Double-blind, Placebo Parallel-controlled Phase I/II Clinical Trial.

This is another inactivated viral vaccine developed by the Beijing Institute of Biological Products/Sinopharm with a study timeline from April 28, 2020, to November 28, 2021 [42].

A Randomized, Double-Blinded, Placebo-Controlled, Phase Ⅰ/Ⅱ Clinical Trial, to Evaluate the Safety and Immunogenicity of the SARS-CoV-2 Inactivated Vaccine in Healthy Adults Aged 18-59 Years

Sinovac, a Beijing based company, is currently testing its inactivated viral COVID-19 vaccine (PiCoVacc) in a randomized, double-blinded, placebo-controlled Phase I trial involving 144 adults [42].

A Phase I/II Study to Determine Efficacy, Safety, and Immunogenicity of the Candidate Coronavirus Disease (COVID-19) Vaccine ChAdOx1 nCoV-19 in UK Healthy Adult Volunteers.

Researchers at the University of Oxford are testing their ChAdOx1 nCoV-19 vaccine, which uses the SARS-CoV-2 spike protein and an adenovirus vaccine vector on 1102 healthy volunteers. The MenACWY vaccine (licensed for groups A, C, W, and Y meningococcus) is serving as the control [42, 45].

Evaluation of the Safety and Immunogenicity of a SARS-CoV-2 rS (COVID-19) Nanoparticle Vaccine With/Without Matrix-M Adjuvant

This is a recombinant SARs-CoV-2 glycoprotein nanoparticle vaccine, which is being adjuvanted with Matrix M. Its developer is Novavax (Rockville, MD), and the trial timeline is May 6, 2020, to July 31, 2021 [42].

A Multi-site Phase I/II, 2-Part, Dose-Escalation Trial Investigating the Safety and Immunogenicity of Four Prophylactic SARS-CoV-2 RNA Vaccines Against COVID-2019 Using Different Dosing Regimens

This vaccine program is in development by BioNTech and Pfizer. It involves four potential vaccines for testing in Germany on 200 healthy participants. The companies will include people with a higher risk for severe COVID-19 during the second stage of testing. Their four vaccine candidates represent different mRNA formats and target antigens [42, 46].

Phase I Open-label Study to Evaluate the Safety, Tolerability, and Immunogenicity of INO-4800, a Prophylactic Vaccine Against SARS-CoV-2, Administered Intradermally Followed by Electroporation in Healthy Volunteers

Inovio Pharmaceuticals in the US began testing this DNA platform vaccine in April; this is a DNA plasmid vaccine with electroporation. Its completion date is set for April 2021 [42, 47].

The clinical trial name of the tenth vaccine approved by the WHO for a clinical trial is not currently available on the WHO website, but it is in development by the Institute of Medical Biology, Chinese Academy of Medical Sciences, and is currently approved for Phase I trial [42].

## Conclusions

COVID-19 has affected the world in a way few could have anticipated. Overall, COVID-19 cases are decreasing in the countries that were affected earlier but other parts of the world are now seeing a surge in cases. It is alarming to see soaring numbers in countries with fewer resources as the healthcare setup may come under immense stress. This is a time where a collective effort is required from the international community to tackle this unprecedented crisis. At a national level, strong commitment from governments and support of the public is required to deal with this menace. In terms of new emerging treatments, we have seen more than a dozen new drugs being investigated, with some of them showing encouraging results. We have a rapidly evolving situation as we keep getting regular updates from the ongoing clinical trials and this article may lack some of the very recent updates by the time it gets published. Vaccination against COVID-19 may be the ultimate solution for this crisis and it is encouraging to see progress in this aspect as ten vaccines have been approved for testing in humans in clinical trials. The medical community is heading in the right direction but will keep requiring support from the government and the public.
